# Sir William Osler, Bart., on Some Results of the War

**Published:** 1915-07-10

**Authors:** 


### RESEARCH DEFENCE SOCIETY.

### Sir William Osler, Bart., on Some Results of the War.

The seventh annual general meeting of members and associates of this Society was held at the Royal Society of Medicine on Wednesday last week. In the absence of Lord Lamington, the President, on military duty, the chair was occupied by General the Hon. Sir Reginald Talbot, K.C.B.

The Chairman, in his opening address, spoke feelingly of the loss to the Society caused by the deaths of vice-presidents and members, including Sir Wm. Anson, Sir Samuel Hoare, Lord Rothschild, Sir Richard Solomon, Sir Henry Littlejohn, Dr. Habershon, and Lady Sieveking. He hoped others would be found willing and able adequately to take their places. He was filled with admiration of the marvellous work which had been done by the medical profession in this great war. The work of the Army and Navy medical departments could only be mentioned with feelings of great gratitude; they had successfully overcome unexampled difficulties, though dealing with enormous masses of men. And a great tribute must be paid to the work of the voluntary hospitals in London and the country generally, not forgetting also the Red Cross and St. John Societies. Their reward should be an increased subscription instead of, as had happened in a number of cases, a diminished revenue. The Research Defence Society had, during the past year, done its best to get people, especially soldiers, to understand what preventive treatment of typhoid meant. And never had the Society done such efficient work, or been engaged on an object which more deserved support. By means of lectures and the distribution of literature, great good had been done in countering the attack on anti-typhoid inoculation which had been organised by anti-vivisectionists; and the number of instances in which the facts put forward had caused soldiers, previously against inoculation, to submit to it, was a striking testimony to the success of the Society's efforts. We in Britain had a great reverence for what was called individual liberty; but it was significant that, while we hesitated in this matter, two Republican nations, the United States of America and France, rendered the measure compulsory by a stroke of the pen. Those who tried to persuade the soldier to resist this means of avoiding death by disease were incurring a grave risk, and if at any time their consciences awoke he did not envy them their state of mind. It was not a case of not knowing the facts. He highly eulogised the energy and self-sacrifice of the Hon. Secretary, Mr. Stephen Paget, whose continual lecturing in bad conditions at the Front had resulted in the loss of his voice. Dr. Sandwith, the Hon. Treasurer, had also done great service, and during his illness Sir David Ferrier had generously filled his place. In the same connection he mentioned the work of the Assistant-Secretary, Miss Burgiss-Brown.

Lord Knutsford, in moving the adoption of the report and statement of accounts, said that until this Society was founded it was nobody's business to protect from foul attacks and cruel accusations men of science, who often worked for the pay of a second-rate clerk. Nor, until this Society came into being, was it anyone's function to contradict the wilful misrepresentations of the anti-vivisection people, or to carry on the great work of education on this matter—the Society's chief work. It was necessary to show the good resulting from experiments on animals, but they could be done without cruelty and result in enormous lessening of suffering in both men and animals.

Dr. F. M. Sandwith seconded the resolution, and explained the origin of the Society's activity in furtherance of anti-typhoid inoculation in the Army.

The resolution was unanimously adopted.

Sir William Osler, F.R.S., proposed the re-election of the executive, the officers, and the auditors, and paid a very eloquent tribute to the Society for the work it had accomplished in the seven years of its existence. We had, he said, two definite enemies : the Germans, whom we had thoroughly and finally to beat; and, secondly, we had the foes of our own household—the diseases which had been the accompaniments of all campaigns and had proved to be much more formidable than the enemy's bullets or bayonets. Against this second class of enemy the Society was doing great work. When the great Civil War in America closed fifty years ago we had not positive knowledge as to the cause of any of the great scourges of humanity. Yet in the intervening time we had come to know the causes of the majority of the pestilences and plagues. We knew the specific nature of the seeds of disease and that they bred true. We could grow disease germs outside the body and produce the identical disease with them after many sub-cultivations. We also knew the method of transmission of epidemic diseases. In consequence of that knowledge and the great energy displayed, this was the first time in the history of our Empire that we had been ten months at war without a single wide-spread epidemic having occurred, notwithstanding that we were now dealing with two and a half millions of men. Among all that host there had not been more than a thousand cases of that scourge of armies—typhoid fever—and there were now very few of tetanus. The case was different in regard to sepsis, owing to the conditions which prevailed, but 60 per cent. to 70 per cent. of these wounded and sick soldiers recovered and were able to return to the firing-line. At last the number laid by in consequence of the enemy's missiles exceeded those due to disease, a result which had never been attained before.

Sir Frederick Macmillan seconded, and the motion was carried unanimously.

Dr. Andrew Balfour, C.M.G., gave a short lecture on immunisation by inoculation, and a very instructive epidiascope demonstration of living bacteria, for which he was heartily thanked.

